# Supplementary material for: The Small RNA Universe of Capitella teleta
Source: Front Mol Biosci. 2022 Feb 25;9:802814. doi: 10.3389/fmolb.2022.802814 (PMC8915122; doi:10.3389/fmolb.2022.802814)
Supplement: Supplementary file 1 [file DataSheet1.ZIP › Supplement/known/CAPTEscaffold_291_16147.pdf]

Provisional ID	: CAPTEscaffold_291_16147
Score total	: 850511.7
Score for star read(s)	: 3.9
Score for read counts	: 850506.2
Score for mfe	: 0.8
Score for randfold	: -2.2
Score for cons. seed	: 3
Total read count	: 1668243
Mature read count	: 1667338
Loop read count	: 1
Star read count	: 904

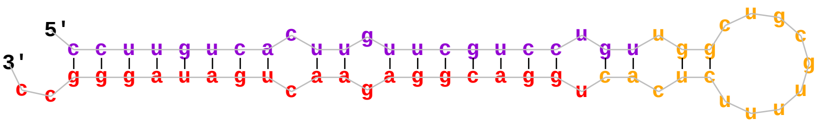

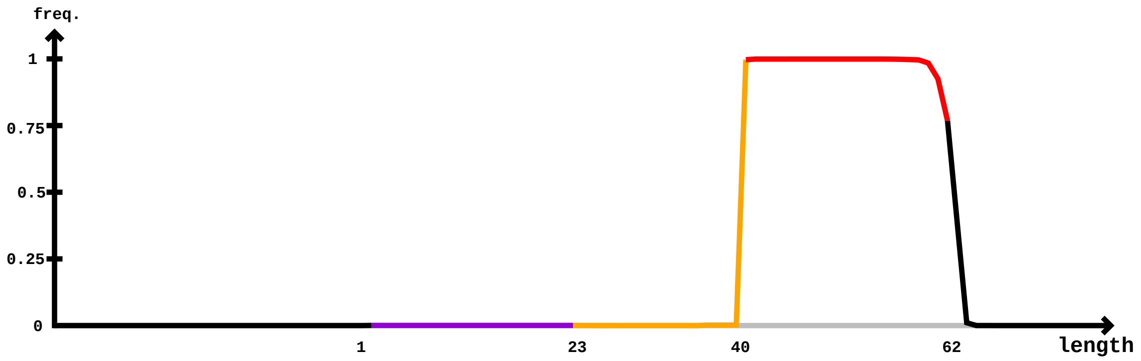

Star **Mature**

5'-	guuaugaagugcguuaugauacaucacguucccuugucacuuguucguccuguuggcugcguuuucucacuggacggagaacugauagggcccugugguggaucacgug	-3'	obs		
	guuaugaagugcguuaugauacaucacguucccuugucacuuguucguccuguuggcugcguuuucucacuggacggagaacugauagggcccugugguggaucacgug		exp		
	guuaugaagugcguuaugauacaucacguucccuugucacuuguucguccuguuggcugcguuuucucacuggacggagaacugauagggcccugugguggaucacgug		known		
	..........((((...((..((.((((...((((((((.((.(((((((.((.((.........)).)).))))))).)).))))))))...))))))..)))))).		reads	mm	sample
	..uGugaagugcguuaugau..		1	1	seq
	...........cguuaugauacaucacguuc...		7	0	seq
	............guuaugauacaucacguuc...		1	0	seq
	.............uuaugauacaucacguuc...		1	0	seq
	.............................ucccuugucacuuguucguccugu...		4	0	seq
	..............................cccuugucacuuguucguccug..		3	0	seq
	...............................ccuugucacuuguucguccu...		1	0	seq
	...............................ccuugucacuuguucguccug..		95	0	seq
	...............................ccuugucacuuguucgucAugu...		2	1	seq
	...............................ccuAgucacuuguucguccugu...		1	1	seq
	...............................ccuugucacuuguucgucUugu...		1	1	seq
	...............................ccuugucacuuguucguccugu...		687	0	seq
	...............................ccuugucacuCguucguccugu...		1	1	seq
	...............................cAuugucacuuguucguccugu...		1	1	seq
	...............................ccuugucacuuguucgCccugu...		1	1	seq
	...............................cUuugucacuuguucguccugu...		1	1	seq
	...............................ccuugucaGuuguucguccugu...		1	1	seq
	...............................ccuugucacuuguucguccugG...		8	1	seq
	...............................ccuugucacuuguucguUcugu...		1	1	seq
	...............................Ucuugucacuuguucguccugu...		1	1	seq
	...............................ccuugucacuuguuUguccugu...		1	1	seq
	...............................ccuugucacuuguucguccuguu..		2	0	seq
	...............................ccuugucacuuguucguccuguuU...		1	1	seq
	...............................ccuugucacuuguucguccuguug...		89	0	seq
	...............................ccuugucacuuguucguccuguuggcu..		1	0	seq
	...............................ccuugucacuuguucguccuguuggcugc...		1	0	seq
	...............................ccuugucacuuguucguccuguuggcugcg..		2	0	seq
	..................................ugucacuuguucguccuguugg..		1	0	seq
	....uggcugcguuuucucacu.....................................		1	0	seq
	....ucacuNgacggagaacuga.......................		1	1	seq
	....ucacAggacggagaacuga.......................		1	1	seq
	....ucacuggacggagaacugG.......................		1	1	seq

Star Mature

```
guuaugaagugcguuaugauacaucacguucccuugucacuuguucguccuguuggcugcguuuucucacuggacggagaacugauagggcccugugugguaucacgug
..................................................................uUacuggacggagaacuga.......................	1	1	seq
..................................................................ucacuggacggagaacuga.......................	167	0	seq
..................................................................ucacuggacggagaUcugau......................	1	1	seq
..................................................................ucacuggacggagaacCgau......................	1	1	seq
..................................................................ucacuggacggagaacugau......................	1146	0	seq
..................................................................ucacuggaUggagaacugau......................	1	1	seq
..................................................................ucacuggacggGgaacugau......................	1	1	seq
..................................................................ucacuAgacggagaacugau......................	5	1	seq
..................................................................uUacuggacggagaacugau......................	2	1	seq
..................................................................Acacuggacggagaacugau......................	9	1	seq
..................................................................ucacuggacAgagaacugau......................	1	1	seq
..................................................................Ncacuggacggagaacugau......................	1	1	seq
..................................................................ucacAggacggagaacugau......................	1	1	seq
..................................................................ucacuggacggagaacAgau......................	1	1	seq
..................................................................uAacuggacggagaacugau......................	1	1	seq
..................................................................ucacuggacAgagaacugaua.....................	3	1	seq
..................................................................uUacuggacggagaacugaua.....................	1	1	seq
..................................................................ucacuggacggagaacugaAa.....................	3	1	seq
..................................................................ucacuggacggagaacuCaua.....................	1	1	seq
..................................................................ucacuggacggagaaAugaua.....................	1	1	seq
..................................................................Gcacuggacggagaacugaua.....................	1	1	seq
..................................................................ucacuggaUggagaacugaua.....................	2	1	seq
..................................................................ucacuggacggagaacuAaua.....................	3	1	seq
..................................................................ucacuggacggGgaacugaua.....................	1	1	seq
..................................................................ucacuggacggagaaUugaua.....................	1	1	seq
..................................................................ucacuggGcggagaacugaua.....................	1	1	seq
..................................................................ucacuAgacggagaacugaua.....................	3	1	seq
..................................................................ucacuggacggagaacugUua.....................	2	1	seq
..................................................................ucacAggacggagaacugaua.....................	3	1	seq
..................................................................ucacuggacggagaacugaua.....................	1652	0	seq
..................................................................Acacuggacggagaacugaua.....................	10	1	seq
..................................................................ucacuggacUgagaacugaua.....................	1	1	seq
..................................................................ucacuggacggagaacugauU.....................	1	1	seq
..................................................................ucacuggacCgagaacugaua.....................	1	1	seq
..................................................................ucacugAacggagaacugaua.....................	1	1	seq
..................................................................ucacuggacggagaacugauaU....................	8	1	seq
..................................................................ucacuggacggagaacugauaC....................	1	1	seq
..................................................................ucacuggacggagaacugauagg...................	1	0	seq
...................................................................cacuggacggagaacugauagU...................	1	1	seq
...................................................................cacuggacggagaacugauagg...................	2	0	seq
....................................................................Gcuggacggagaacugau......................	1	1	seq
....................................................................Ucuggacggagaacugauaggg..................	1	1	seq
....................................................................Ccuggacggagaacugauagggcc................	2	1	seq
....................................................................Ucuggacggagaacugauagggcc................	6	1	seq
....................................................................acuggacggagaacugauagggcc................	1	0	seq
....................................................................acuggacggagaacugauagggccU...............	1	1	seq
.....................................................................cuggacggagaacugauagg...................	3	0	seq
.....................................................................Auggacggagaacugauagg...................	1	1	seq
.....................................................................cuggacggagaacugauaggg..................	11	0	seq
.....................................................................cuggacggagaacugauagggc.................	17	0	seq
.....................................................................Guggacggagaacugauagggc.................	2	1	seq
.....................................................................cuggacggagaacugauagggcc................	28	0	seq
.....................................................................Uuggacggagaacugauagggcc................	1	1	seq
.....................................................................cuggacggagaacugaAagggcc................	1	1	seq
.....................................................................Guggacggagaacugauagggcc................	10	1	seq
.....................................................................cuggacggagaacugauagggccU...............	23	1	seq
.....................................................................cuggacggagaacugauagggccUu..............	1	1	seq
......................................................................uggaUggagaacugauag....................	1	1	seq
......................................................................uggacggagaacugGuag....................	2	1	seq
......................................................................uggCcggagaacugauag....................	1	1	seq
......................................................................uggacggagaacuAauag....................	1	1	seq
......................................................................ugAacggagaacugauag....................	1	1	seq
......................................................................uAgacggagaacugauag....................	6	1	seq
......................................................................uggacCgagaacugauag....................	1	1	seq
......................................................................uggacggagaacuUauag....................	1	1	seq
......................................................................uUgacggagaacugauag....................	2	1	seq
......................................................................uggacggagaacugauaC....................	4	1	seq
......................................................................uggacAgagaacugauag....................	2	1	seq
......................................................................uggacggagaaGugauag....................	1	1	seq
......................................................................uggacggaUaacugauag....................	1	1	seq
```

```
                               Star                                     Mature
guuaugaagugcguuaugauacaucacguucccuugucacuuguucguccuguuggcugcguuuucucacuggacggagaacugauagggcccugugugguaucacgug
......................................................................uggaGggagaacugauag.....................  1      1  seq
......................................................................uggacggagaacugaAag.....................  6      1  seq
......................................................................Aggacggagaacugauag.....................  8      1  seq
......................................................................Nggacggagaacugauag.....................  4      1  seq
......................................................................uggacggagaaAugauag.....................  1      1  seq
......................................................................Gggacggagaacugauag.....................  1      1  seq
......................................................................uggacggagaacCgauag.....................  1      1  seq
......................................................................uggacggagaacugauaU.....................  77     1  seq
......................................................................uggacggagaaUugauag.....................  1      1  seq
......................................................................uggacgAagaacugauag.....................  2      1  seq
......................................................................ugCacggagaacugauag.....................  1      1  seq
......................................................................uggacggUgaacugauag.....................  1      1  seq
......................................................................uggacggagaacugauag.....................  2174   0  seq
......................................................................uggacggagaacugauagA....................  5464   1  seq
......................................................................Cggacggagaacugauagg....................  1      1  seq
......................................................................Gggacggagaacugauagg....................  11     1  seq
......................................................................uggacAgagaacugauagg....................  15     1  seq
......................................................................uggacggagGacugauagg....................  3      1  seq
......................................................................uggGcggagaacugauagg....................  6      1  seq
......................................................................Aggacggagaacugauagg....................  52     1  seq
......................................................................uggaAggagaacugauagg....................  1      1  seq
......................................................................Nggacggagaacugauagg....................  3      1  seq
......................................................................uggacgUagaacugauagg....................  1      1  seq
......................................................................uggacggagaacugauGgg....................  2      1  seq
......................................................................uggacggagaacugauagU....................  593    1  seq
......................................................................uggacggagUacugauagg....................  1      1  seq
......................................................................uCgacggagaacugauagg....................  5      1  seq
......................................................................uggacggGgaacugauagg....................  1      1  seq
......................................................................uggacggagaacugGuagg....................  2      1  seq
......................................................................uAgacggagaacugauagg....................  24     1  seq
......................................................................uggacggagaGcugauagg....................  3      1  seq
......................................................................uggacggagaacugauagC....................  14     1  seq
......................................................................uggacggagaacugauagg....................  13424  0  seq
......................................................................uggacggagaacugauUgg....................  1      1  seq
......................................................................uggacggagaCcugauagg....................  1      1  seq
......................................................................uggacCgagaacugauagg....................  1      1  seq
......................................................................uggacggagaacugauaUg....................  28     1  seq
......................................................................uggacggagaaAugauagg....................  3      1  seq
......................................................................uggacggagaacAgauagg....................  2      1  seq
......................................................................uggacggagaacuAauagg....................  11     1  seq
......................................................................uggacggagaacugaAagg....................  60     1  seq
......................................................................uUgacggagaacugauagg....................  1      1  seq
......................................................................uggacggagaacugUuagg....................  2      1  seq
......................................................................uggacgAagaacugauagg....................  2      1  seq
......................................................................uggacggaAaacugauagg....................  1      1  seq
......................................................................uggaUggagaacugauagg....................  3      1  seq
......................................................................uggCcggagaacugauagg....................  1      1  seq
......................................................................uggUcggagaacugauagg....................  2      1  seq
......................................................................uggacggagaacCgauagg....................  2      1  seq
......................................................................ugCacggagaacugauagg....................  3      1  seq
......................................................................uggacUgagaacugauagg....................  1      1  seq
......................................................................uggacggaUaacugauagg....................  1      1  seq
......................................................................uggacggUgaacugauagg....................  3      1  seq
......................................................................ugAacggagaacugauagg....................  3      1  seq
......................................................................Gggacggagaacugauaggg...................  60     1  seq
......................................................................uggacAgagaacugauaggg...................  171    1  seq
......................................................................uCgacggagaacugauaggg...................  7      1  seq
......................................................................Aggacggagaacugauaggg...................  368    1  seq
......................................................................uggacggagaaAugauaggg...................  25     1  seq
......................................................................uggacggagaacuCauaggg...................  4      1  seq
......................................................................uAgacggagaacugauaggg...................  256    1  seq
......................................................................uggacCgagaacugauaggg...................  8      1  seq
......................................................................uggacggagaacugaGaggg...................  8      1  seq
......................................................................uggacggagaacAgauaggg...................  27     1  seq
......................................................................uggCcggagaacugauaggg...................  5      1  seq
......................................................................uggacgCagaacugauaggg...................  12     1  seq
......................................................................uggacggagaaUugauaggg...................  5      1  seq
......................................................................uggacggagaacugauaggU...................  154    1  seq
......................................................................uggacggagaacugauCggg...................  1      1  seq
......................................................................uggacNgagaacugauaggg...................  2      1  seq
```

Star **Mature**

guuaugaagugcguuaugauacaucacguuc**ccuugucacuuguucguccugu**uggcugcguuuucucac**uggacggagaacugauagggcc**cuguguggaucacgug

....uggacggagaGcugauaggg..................	18	1	seq
....uggacggaUaacugauaggg..................	5	1	seq
....uggacggagaacuUauaggg..................	6	1	seq
....ugAacggagaacugauaggg..................	26	1	seq
....uggGcggagaacugauaggg..................	7	1	seq
....uggacggaCaacugauaggg..................	6	1	seq
....uggacggagaacuAauaggg..................	65	1	seq
....uUgacggagaacugauaggg..................	9	1	seq
....uggacggagaaGugauaggg..................	1	1	seq
....uggacggagaacugGuaggg..................	4	1	seq
....uggacggagaacugauaggC..................	19	1	seq
....uggacggGgaacugauaggg..................	29	1	seq
....uggaUggagaacugauaggg..................	26	1	seq
....Cggacggagaacugauaggg..................	11	1	seq
....uggacgUagaacugauaggg..................	12	1	seq
....uggacggagaacugaAaggg..................	87	1	seq
....uggacggagaacugauaggA..................	1812	1	seq
....uggacggagaacugauGggg..................	13	1	seq
....uggaGggagaacugauaggg..................	3	1	seq
....uggacggaAaacugauaggg..................	5	1	seq
....ugCacggagaacugauaggg..................	20	1	seq
....ugUacggagaacugauaggg..................	10	1	seq
....uggacggagaacCgauaggg..................	12	1	seq
....uggacggagaacugauaUgg..................	66	1	seq
....uggacggagaacugauagCg..................	6	1	seq
....uggacgAagaacugauaggg..................	25	1	seq
....uggacggagaacugauUggg..................	10	1	seq
....uggacggagUacugauaggg..................	6	1	seq
....uggacggagaacugauagAg..................	75	1	seq
....uggaAggagaacugauaggg..................	14	1	seq
....uggacggagaacugUuaggg..................	5	1	seq
....uggacggagaUcugauaggg..................	8	1	seq
....uggacggUgaacugauaggg..................	17	1	seq
....uNgacggagaacugauaggg..................	2	1	seq
....uggacggagaacugaCaggg..................	14	1	seq
....uggacggagaacugauaCgg..................	21	1	seq
....uggacggagaacugCuaggg..................	1	1	seq
....uggUcggagaacugauaggg..................	15	1	seq
....uggacggagaacugauagUg..................	15	1	seq
....uggacggagGacugauaggg..................	18	1	seq
....uggacUgagaacugauaggg..................	8	1	seq
....uggacggagNacugauaggg..................	1	1	seq
....uggacggagaacugauaggg..................	94709	0	seq
....Nggacggagaacugauaggg..................	22	1	seq
....uggacggagaacGgauaggg..................	3	1	seq
....uggaUggagaacugauagggc.................	41	1	seq
....Gggacggagaacugauagggc.................	180	1	seq
....uggacggagaCcugauagggc.................	2	1	seq
....uggacgAagaacugauagggc.................	37	1	seq
....uggacggagaacugaGagggc.................	13	1	seq
....uggacggagaacugauagggU.................	2248	1	seq
....uggacggagaacugauaggAc.................	32	1	seq
....uggacggagaacuCauagggc.................	12	1	seq
....uggacggagaacugauaAggc.................	966	1	seq
....uggacggagaacugauaggCc.................	135	1	seq
....uggacggagaUcugauagggc.................	25	1	seq
....Nggacggagaacugauagggc.................	65	1	seq
....uggacggagaacugaCagggc.................	19	1	seq
....uggacggagaacGgauagggc.................	11	1	seq
....uggacggagaacugauaggUc.................	27	1	seq
....uggUcggagaacugauagggc.................	29	1	seq
....Aggacggagaacugauagggc.................	854	1	seq
....uggacggagaacugauCgggc.................	2	1	seq
....uggacggGgaacugauagggc.................	42	1	seq
....uggacggagaacCgauagggc.................	34	1	seq
....uggCcggagaacugauagggc.................	15	1	seq
....ugUacggagaacugauagggc.................	25	1	seq
....uggacggagaacugauUgggc.................	11	1	seq
....ugCacggagaacugauagggc.................	64	1	seq
....uggacggagaacugauaUggc.................	18	1	seq

Star Mature

```
guuaugaagugcguuaugauacaucacguuc ccuugucacuuguucguccugu uggcugcguuuucucac uggacggagaacugauagggcc cugugugg aucacgug
```

```
guuaugaagugcguuaugauacaucacguucccuugucacuuguucguccuguuggcugcguuuucucacuggacggagaacugauagggcccuguguggaucacgug
......................................................................uggacggaAaacugauagggc.................   27      1   seq
......................................................................uggacggagaacugauagAgc.................   145     1   seq
......................................................................uggacggagaacugauagUgc.................   18      1   seq
......................................................................uggacCgagaacugauagggc.................   18      1   seq
......................................................................uggacggagaaGugauagggc.................   9       1   seq
......................................................................uggacggagaacAgauagggc.................   69      1   seq
......................................................................uggacggUgaacugauagggc.................   47      1   seq
......................................................................uggacggagaacugaAagggc.................   370     1   seq
......................................................................uggacgUagaacugauagggc.................   26      1   seq
......................................................................uggacggagaacugauagggG.................   266     1   seq
......................................................................uggacNgagaacugauagggc.................   7       1   seq
......................................................................uggacggagaacugUuagggc.................   30      1   seq
......................................................................uggacggagaacugGuagggc.................   31      1   seq
......................................................................uNgacggagaacugauagggc.................   6       1   seq
......................................................................uggacggagaaUugauagggc.................   39      1   seq
......................................................................ugAacggagaacugauagggc.................   43      1   seq
......................................................................uggacggagaacugauagggA.................   11477   1   seq
......................................................................uggaAggagaacugauagggc.................   34      1   seq
......................................................................uAgacggagaacugauagggc.................   671     1   seq
......................................................................uggacggagaacugauagggc.................   243350  0   seq
......................................................................uggacAgagaacugauagggc.................   462     1   seq
......................................................................Cggacggagaacugauagggc.................   41      1   seq
......................................................................uggacggagaacugauagggN.................   3       1   seq
......................................................................uggacgCagaacugauagggc.................   17      1   seq
......................................................................uggacggCgaacugauagggc.................   3       1   seq
......................................................................uggGcggagaacugauagggc.................   46      1   seq
......................................................................uggacggagaacuAauagggc.................   188     1   seq
......................................................................uggacggagUacugauagggc.................   27      1   seq
......................................................................uggacggagaacugauaCggc.................   15      1   seq
......................................................................uggacggaUaacugauagggc.................   8       1   seq
......................................................................uggacggagaGcugauagggc.................   54      1   seq
......................................................................uggacggagaaAugauagggc.................   45      1   seq
......................................................................uggacggagaacugauagCgc.................   15      1   seq
......................................................................uggaGggagaacugauagggc.................   6       1   seq
......................................................................uggacggagCacugauagggc.................   2       1   seq
......................................................................uggacggagaacuUauagggc.................   21      1   seq
......................................................................uUgacggagaacugauagggc.................   21      1   seq
......................................................................uggacggagaacugauGgggc.................   36      1   seq
......................................................................uCgacggagaacugauagggc.................   32      1   seq
......................................................................uggacggagGacugauagggc.................   38      1   seq
......................................................................uggacUgagaacugauagggc.................   32      1   seq
......................................................................uggacggaCaacugauagggc.................   14      1   seq
......................................................................uggacggagaacugaCagggcc................   166     1   seq
......................................................................uggacggagaacugauagCgcc................   53      1   seq
......................................................................uCgacggagaacugauagggcc................   153     1   seq
......................................................................uggacggagaacugauagggcN................   2       1   seq
......................................................................uggacggagaacugCuagggcc................   4       1   seq
......................................................................uggacggagaacCgauagggcc................   183     1   seq
......................................................................ugCacggagaacugauagggcc................   226     1   seq
......................................................................uggacggagaacugUuagggcc................   95      1   seq
......................................................................uggacggagaacugauaCggcc................   56      1   seq
......................................................................uggacggagaaAugauagggcc................   164     1   seq
......................................................................uggacggagaGcugauagggcc................   152     1   seq
......................................................................uggacggagGacugauagggcc................   130     1   seq
......................................................................uggacggGgaacugauagggcc................   199     1   seq
......................................................................uggacggagaacAgauagggcc................   271     1   seq
......................................................................Aggacggagaacugauagggcc................   3013    1   seq
......................................................................Nggacggagaacugauagggcc................   257     1   seq
......................................................................uggacggagaNcugauagggcc................   1       1   seq
......................................................................uggacggagNacugauagggcc................   1       1   seq
......................................................................uggacggagaacugauagggcG................   159     1   seq
......................................................................uggacggagCacugauagggcc................   11      1   seq
......................................................................uggacggagaCcugauagggcc................   10      1   seq
......................................................................uggacggagaacugaNagggcc................   1       1   seq
......................................................................uggacggaCaacugauagggcc................   55      1   seq
......................................................................ugAacggagaacugauagggcc................   221     1   seq
......................................................................uggacggagaacugauaggUcc................   87      1   seq
......................................................................uggacggagaacugauaAggcc................   1104    1   seq
......................................................................uggacggaUaacugauagggcc................   43      1   seq
......................................................................uggaAggagaacugauagggcc................   144     1   seq
```

```
                                               Star                                   Mature
guuaugaagugcguuaugauacaucacguucccuugucacuuguucguccuguuggcugcguuuucucacuggacggagaacugauagggcccuguguggaucacgug
......................................................................uggacggagaacugaAagggcc................   510      1    seq
......................................................................uNgacggagaacugauagggcc................   27       1    seq
......................................................................uggacggagUacugauagggcc................   102      1    seq
......................................................................uggacggagaacugauagggAc................   277      1    seq
......................................................................uUgacggagaacugauagggcc................   100      1    seq
......................................................................uggacgCagaacugauagggcc................   85       1    seq
......................................................................uggacggagaacugauaggNcc................   1        1    seq
......................................................................uggacggagaaGugauagggcc................   23       1    seq
......................................................................uggGcggagaacugauagggcc................   168      1    seq
......................................................................uggacggagaacugauagggNc................   5        1    seq
......................................................................uggacggaAaacugauagggcc................   137      1    seq
......................................................................ugUacggagaacugauagggcc................   90       1    seq
......................................................................uggacggagaacugauaUggcc................   84       1    seq
......................................................................uggacggagaacugauUgggcc................   80       1    seq
......................................................................uggacAgagaacugauagggcc................   2251     1    seq
......................................................................uggacggagaacugauagUgcc................   38       1    seq
......................................................................uggUcggagaacugauagggcc................   142      1    seq
......................................................................uggacggagaacuUauagggcc................   93       1    seq
......................................................................uggacggagaacGgauagggcc................   53       1    seq
......................................................................uggacggagaacugauGgggcc................   121      1    seq
......................................................................uggacggUgaacugauagggcc................   132      1    seq
......................................................................uggacNgagaacugauagggcc................   13       1    seq
......................................................................uggacggagaacugauagggcA................   7953     1    seq
......................................................................uggacgAagaacugauagggcc................   126      1    seq
......................................................................uggacggagaacugauagggcc................   951817   0    seq
......................................................................uggaUggagaacugauagggcc................   185      1    seq
......................................................................uggacggagaacugauaggAcc................   134      1    seq
......................................................................uAgacggagaacugauagggcc................   3215     1    seq
......................................................................uggacggagaacugauCgggcc................   9        1    seq
......................................................................uggacggagaacuAauagggcc................   1062     1    seq
......................................................................uggacUgagaacugauagggcc................   133      1    seq
......................................................................uggacgUagaacugauagggcc................   111      1    seq
......................................................................uggacggagaacuCauagggcc................   58       1    seq
......................................................................uggacggagaacugauagAgcc................   499      1    seq
......................................................................Gggacggagaacugauagggcc................   783      1    seq
......................................................................uggacggagaUcugauagggcc................   119      1    seq
......................................................................uggacggaNaacugauagggcc................   1        1    seq
......................................................................uggacggagaaUugauagggcc................   107      1    seq
......................................................................ugNacggagaacugauagggcc................   5        1    seq
......................................................................uggacggagaacugauagggGc................   1194     1    seq
......................................................................uggacggagaacugauagggUc................   141      1    seq
......................................................................uggaGggagaacugauagggcc................   32       1    seq
......................................................................uggacggagaacugaGagggcc................   23       1    seq
......................................................................Cggacggagaacugauagggcc................   225      1    seq
......................................................................uggCcggagaacugauagggcc................   63       1    seq
......................................................................uggacCgagaacugauagggcc................   94       1    seq
......................................................................uggacggagaacugauaggCcc................   67       1    seq
......................................................................uggacggCgaacugauagggcc................   6        1    seq
......................................................................uggacggagaacugGuagggcc................   120      1    seq
......................................................................uggacggagaacugauagggcU................   9553     1    seq
......................................................................uAgacggagaacugauagggccc...............   9        1    seq
......................................................................uggacggagaacuUauagggccc...............   1        1    seq
......................................................................uggacggagaacugauagAgccc...............   2        1    seq
......................................................................Aggacggagaacugauagggccc...............   5        1    seq
......................................................................uggacggagaacugauagggcGc...............   1        1    seq
......................................................................uggacggagaacugauagggccA...............   56627    1    seq
......................................................................uggacggagaacugauagggccU...............   212609   1    seq
......................................................................uggacggagaaUugauagggccc...............   1        1    seq
......................................................................ugAacggagaacugauagggccc...............   1        1    seq
......................................................................uggacAgagaacugauagggccc...............   7        1    seq
......................................................................uggacggagaacugauagggccG...............   2624     1    seq
......................................................................uggacggagaacugauagggcAc...............   10       1    seq
......................................................................uggacggagaacuAauagggccc...............   3        1    seq
......................................................................uggacggagaacugauagggccc...............   1019     0    seq
......................................................................uggacggagUacugauagggccc...............   1        1    seq
......................................................................uggacggagaacugauagggccN...............   4        1    seq
......................................................................uggacggagaacugauagggcUc...............   15       1    seq
......................................................................uggacggagaacugauagAgcccu..............   1        1    seq
......................................................................uggacggagaacugauagggccUu..............   10262    1    seq
......................................................................Aggacggagaacugauagggcccu..............   1        1    seq
```

```
                                   Star                                 Mature
guuaugaagugcguuaugauacaucacguucccuugucacuuguucguccuguuggcugcguuuucucacuggacggagaacugauagggcccuguguggaucacgug
......................................................................uggacggagaacugauagggcUcu..............   1      1    seq
......................................................................uggacggagaacugauagggcccC..............   3      1    seq
......................................................................uggacggaUaacugauagggcccu..............   1      1    seq
......................................................................uggacggagaacugauagggccAu..............   6609   1    seq
......................................................................Nggacggagaacugauagggcccu..............   1      1    seq
......................................................................uggacggagaacugauagggcccG..............   1      1    seq
......................................................................uggacAgagaacugauagggcccu..............   1      1    seq
......................................................................uggacggagaacugaAagggcccu..............   1      1    seq
......................................................................uAgacggagaacugauagggcccu..............   2      1    seq
......................................................................uggacggagaacugauagggcccA..............   98     1    seq
......................................................................uggacggagaacugauagggccGu..............   4      1    seq
......................................................................uggacggagaacugauagggcccu..............   367    0    seq
......................................................................uggacggagaacugauagggccUug.............   5      1    seq
......................................................................uggacggagaacugauagggcccuU.............   40     1    seq
......................................................................uggacggagaacugauagggcccuC.............   1      1    seq
......................................................................uggacggagaacugauagggccAug.............   1      1    seq
......................................................................uggacggagaacugauagggcccuA.............   6      1    seq
......................................................................uggacggagaacugauagggcccuUu............   40     1    seq
......................................................................uggacggagaacugauagggccAugu............   95     1    seq
......................................................................uggacggagaacugauagggcccuAu............   29     1    seq
......................................................................uggacggagaacugauagggccUugu............   20     1    seq
.......................................................................ggacggagaacugauagA...................   13     1    seq
.......................................................................ggGcggagaacugauagg...................   1      1    seq
.......................................................................Ugacggagaacugauagg...................   1      1    seq
.......................................................................ggacggagaacugauagg...................   21     0    seq
.......................................................................ggacggagaacugauaggg..................   317    0    seq
.......................................................................ggacggagaacugauaggA..................   1      1    seq
.......................................................................ggacggagaacAgauaggg..................   1      1    seq
.......................................................................ggacggagaacugauUggg..................   1      1    seq
.......................................................................ggacggagaacugauagAg..................   2      1    seq
.......................................................................ggGcggagaacugauaggg..................   11     1    seq
.......................................................................ggacggagCacugauaggg..................   1      1    seq
.......................................................................ggacggagaacugauaUgg..................   1      1    seq
.......................................................................ggacggagaacGgauaggg..................   1      1    seq
.......................................................................ggacggagaaAugauaggg..................   2      1    seq
.......................................................................Ugacggagaacugauaggg..................   9      1    seq
.......................................................................ggacAgagaacugauaggg..................   1      1    seq
.......................................................................ggacggagaacugauaAggc.................   4      1    seq
.......................................................................ggacggagaacugauagggU.................   3      1    seq
.......................................................................ggacggagaacugauagggA.................   16     1    seq
.......................................................................ggacggagaacugaCagggc.................   1      1    seq
.......................................................................ggacgAagaacugauagggc.................   1      1    seq
.......................................................................ggacggaAaacugauagggc.................   1      1    seq
.......................................................................ggacggagCacugauagggc.................   5      1    seq
.......................................................................ggacggagaacugauagggG.................   1      1    seq
.......................................................................ggacggagaacAgauagggc.................   3      1    seq
.......................................................................ggacggagaacugauagggc.................   521    0    seq
.......................................................................Ugacggagaacugauagggc.................   20     1    seq
.......................................................................ggGcggagaacugauagggc.................   20     1    seq
.......................................................................ggacggagaaAugauagggcc................   3      1    seq
.......................................................................Ngacggagaacugauagggcc................   1      1    seq
.......................................................................ggacggagGacugauagggcc................   3      1    seq
.......................................................................ggacggagaacugauagggcc................   2091   0    seq
.......................................................................ggacggagaacugauagggcA................   15     1    seq
.......................................................................ggacggaUaacugauagggcc................   1      1    seq
.......................................................................ggacggagaacugauagggGc................   1      1    seq
.......................................................................ggacAgagaacugauagggcc................   1      1    seq
.......................................................................ggacggagaacAgauagggcc................   22     1    seq
.......................................................................ggacggagaGcugauagggcc................   1      1    seq
.......................................................................ggacggagaacugauagggcU................   10     1    seq
.......................................................................ggacggagaacugaCagggcc................   1      1    seq
.......................................................................ggacggagUacugauagggcc................   2      1    seq
.......................................................................ggacggagaUcugauagggcc................   1      1    seq
.......................................................................ggacggagaacugauagggUc................   2      1    seq
.......................................................................ggacCgagaacugauagggcc................   1      1    seq
.......................................................................ggacggagaacugaAagggcc................   5      1    seq
.......................................................................ggGcggagaacugauagggcc................   121    1    seq
.......................................................................ggacggagCacugauagggcc................   9      1    seq
.......................................................................ggacggagaacugauaAggcc................   2      1    seq
.......................................................................Ugacggagaacugauagggcc................   71     1    seq
```

Star **Mature**

guuaugaagugcguuaugauacaucacguucccuugucacuuguucguccuguuggcugcguuuucucacuggacggagaacugauagggcccuguguggaucacgug			
....ggacggagaacuUauagggcc................	1	1	seq
....ggacgAagaacugauagggcc................	1	1	seq
....ggacggagaacugauaUggcc................	6	1	seq
....ggacggagaacCgauagggcc................	1	1	seq
....ggacggagaacugauagggcG................	2	1	seq
....ggacggagaacugauagggccA...............	130	1	seq
....ggacggagaacugauagggccU...............	500	1	seq
....ggacggagaacugauagggccc...............	3	0	seq
....ggacggagaacugauagggccG...............	5	1	seq
....ggacggagaacugauagggccAu..............	55	1	seq
....ggacggagaacugauagggccUu..............	29	1	seq
....ggacggagaacugauagggcccu..............	1	0	seq
....gacggagaacugauaggg..................	2	0	seq
....Cacggagaacugauagggcc................	1	1	seq
....Uacggagaacugauagggcc................	3	1	seq
....gacggagaacugauagggcc................	16	0	seq
....gacggagaacugauagggccU...............	2	1	seq
....gacggagaacugauagggccA...............	3	1	seq
....acggagaacugauagggc.................	2	0	seq
....acggagaacCgauagggcc................	1	1	seq
....Gcggagaacugauagggcc................	1	1	seq
....acggagaacugauagggcc................	37	0	seq
....acggagaacugauagggccG...............	1	1	seq
....acggagaacugauagggccA...............	3	1	seq
....acggagaacugauagggccU...............	5	1	seq
....cAgagaacugauagggcc................	1	1	seq
....Uggagaacugauagggcc................	9	1	seq
....cggagaacugauagggcc................	19	0	seq
....cggagaacugauagggccA...............	3	1	seq
....cggagaacugauagggccU...............	9	1	seq
....ggagaacugauagggccA...............	13	1	seq
....ggagaacugauagggccU...............	5	1	seq
....gagaacugauagggccUu..............	1	1	seq
